# 6-Ferrocenoyl-7-(4-fluoro­phen­yl)spiro­[hexa­hydro­pyrrolo­[1,2-*c*][1,3]thia­zole-5,11′-indeno­[1,2-*b*]quinoxaline]

**DOI:** 10.1107/S1600536813022617

**Published:** 2013-08-17

**Authors:** Sivasubramanian Suhitha, Krishnaswamy Gunasekaran, Deivasigamani Gavaskar, Raghavachary Raghunathan, Devadasan Velmurugan

**Affiliations:** a Centre of Advanced Study in Crystallography and Biophysics, University of Madras, Guindy Campus, Chennai 600 025, India; b Department of Organic Chemistry, University of Madras, Guindy Campus, Chennai 600 025, India

## Abstract

In the title compound, [Fe(C_5_H_5_)(C_32_H_23_FN_3_OS)], both the thia­zolidine ring and the pyrrolidine ring adopt a twist conformation on the N—C(H) bridging bond. Their mean planes are inclined to one another by 10.05 (10)°, and they make dihedral angles of 82.09 (10) and 89.67 (11)°, respectively, with the cyclo­pentane ring. The F atom deviates by −0.0238 (2) Å from the benzene ring to which it is attached. In the crystal, mol­ecules are linked by a pair of C—H⋯O hydrogen bonds, forming inversion dimers.

## Related literature

For the biological activity of ferrocene derivatives, see: Jaouen *et al.* (2004 ▶); Biot *et al.* (2004 ▶); Fouda *et al.* (2007 ▶). For a related structure, see: Vijayakumar *et al.* (2012 ▶).
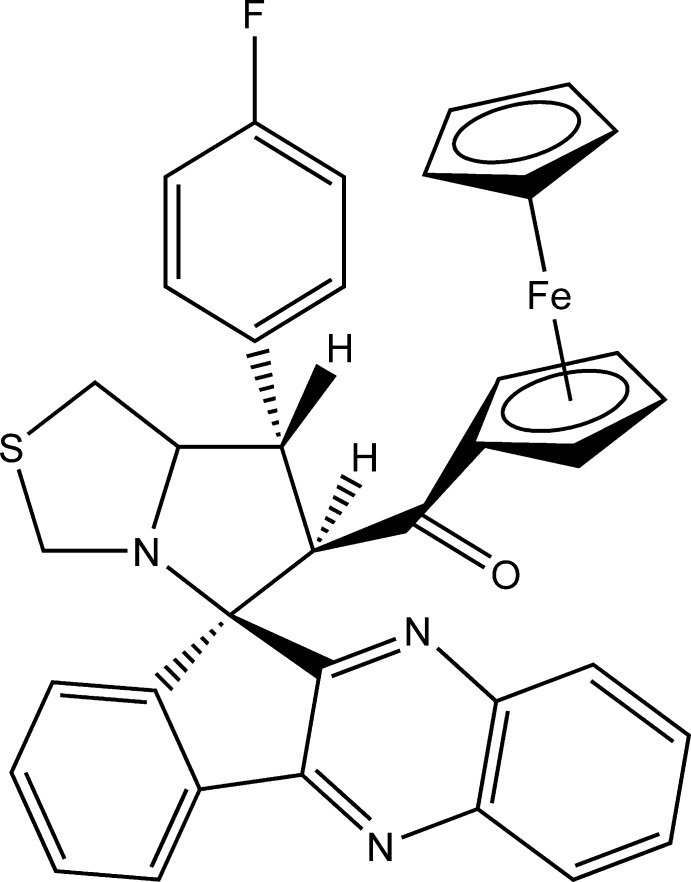


## Experimental

### 

#### Crystal data

[Fe(C_5_H_5_)(C_32_H_23_FN_3_OS)]
*M* *_r_* = 637.53
Triclinic, 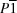

*a* = 8.7097 (2) Å
*b* = 12.6456 (3) Å
*c* = 13.5477 (4) Å
α = 83.865 (1)°
β = 79.008 (1)°
γ = 86.776 (1)°
*V* = 1455.39 (6) Å^3^
*Z* = 2
Mo *K*α radiation
μ = 0.63 mm^−1^
*T* = 293 K
0.30 × 0.25 × 0.20 mm

#### Data collection

Bruker SMART APEXII area-detector diffractometer
Absorption correction: multi-scan (*SADABS*; Bruker, 2008 ▶) *T*_min_ = 0.833, *T*_max_ = 0.884
22064 measured reflections
5966 independent reflections
5126 reflections with *I* > 2σ(*I*)
*R*_int_ = 0.022

#### Refinement

*R*[*F* ^2^ > 2σ(*F* ^2^)] = 0.035
*wR*(*F* ^2^) = 0.097
*S* = 1.06
5966 reflections
397 parameters
H-atom parameters constrained
Δρ_max_ = 0.33 e Å^−3^
Δρ_min_ = −0.34 e Å^−3^

### 

Data collection: *APEX2* (Bruker, 2008 ▶); cell refinement: *SAINT* (Bruker, 2008 ▶); data reduction: *SAINT*; program(s) used to solve structure: *SHELXS97* (Sheldrick, 2008 ▶); program(s) used to refine structure: *SHELXL97* (Sheldrick, 2008 ▶); molecular graphics: *ORTEP-3 for Windows* (Farrugia, 2012 ▶); software used to prepare material for publication: *SHELXL97* and *PLATON* (Spek, 2009 ▶).

## Supplementary Material

Crystal structure: contains datablock(s) global, I. DOI: 10.1107/S1600536813022617/su2635sup1.cif

Structure factors: contains datablock(s) I. DOI: 10.1107/S1600536813022617/su2635Isup2.hkl

Additional supplementary materials:  crystallographic information; 3D view; checkCIF report

## Figures and Tables

**Table 1:** Hydrogen-bond geometry (Å, °)

*D*—H⋯*A*	*D*—H	H⋯*A*	*D*⋯*A*	*D*—H⋯*A*
C25—H25⋯O1^i^	0.93	2.54	3.212 (2)	129

Symmetry code: (i) 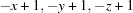.

**Fig. 1.**
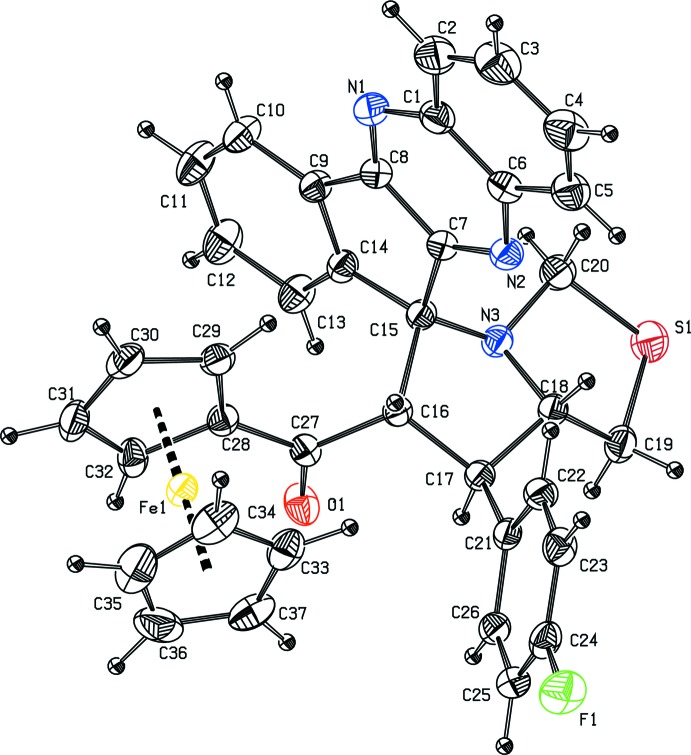
The molecular structure of the title molecule, with atom labelling. Displacement ellipsoids are drawn at the 30% probability level.

**Fig. 2.**
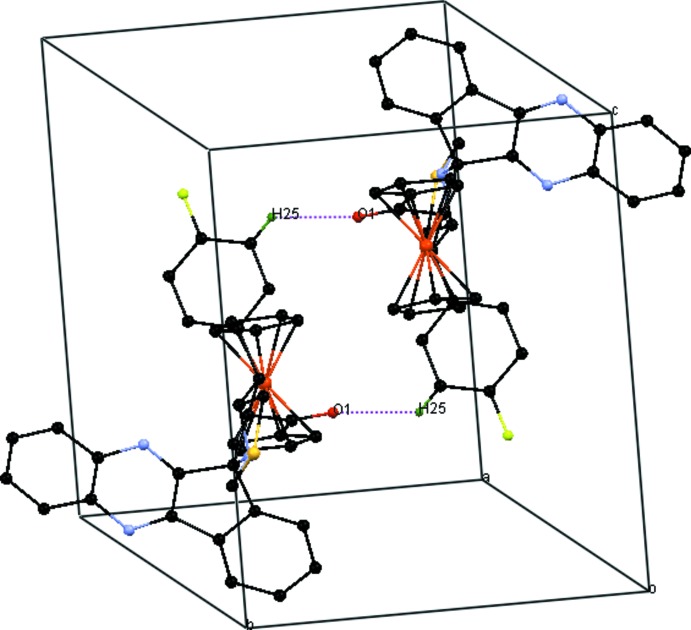
The crystal packing of the title compound viewed along the a axis. The C-H···O hydrogen bonds are shown as dashed lines (see Table 1 for details; H-atoms not involved in hydrogen bonding have been omitted for clarity).

## supplementary crystallographic information

### 1. Comment

Ferrocene attached compounds are well known to have biological activities, such as antimalarial, antifungal (Biot *et al.*, 2004), antitumor (Jaouen *et al.*, 2004), and antibacterial (Fouda *et al.*, 2007). Against this background, and in order to gain information on the molecular conformations and crystal packing, we report herein on the synthesis and crystal structure of the title compound.

In the title compound, Fig. 1, both the thiazolidine ring and the pyrrolidine ring adopt a *twist* conformation on bond N3-C18. The thiazolidine ring (S1/N3/C18-C20) mean plane makes a dihedral angle of 10.05 (10)° with the pyrrolidine ring (N3/C15-C18) mean plane, it also makes a dihedral angle of 82.09 (10)° with the cyclopentane ring (C7-C9/C14/C15) which shows that they are almost perpendicular to each other. The pyrrolidine ring mean plane makes a dihedral angle of 89.67 (11)° with the cyclopentane ring which shows they too are almost orthogonal to each other. The fluorine atom F1 attached with the phenyl ring (C21-C26) deviates by -0.0238 (2)Å.

In the crystal, molecules are linked by a pair of C-H···O hydrogen bonds forming inversion dimers (Table 1 and Fig. 2).

### 2. Experimental

Ninhydrin (1 mmol) and 1, 2-phenylenediamine (1 mmol) were mixed and stirred with 10 ml of methanol for 10 min. To this mixture 1 mmol of thioproline and 1-ferrocenyl-3-(4-fluoro) phenyl prop-2-ene-1-one dipolarophile were added and the mixture was refluxed up to the end of the reaction as observed by TLC. The solvent content from the mixture was removed under reduced pressure and the crude product was obtained. Using column chromatography the crude extract was purified with a 4:1 ratio of petroleum ether and ethyl acetate. Finally, single crystals suitable for the X-ray diffraction were obtained by slow evaporation of the solvent at room temperature.

### 3. Refinement

Hydrogen atoms were placed in calculated positions with C—H ranging from 0.93 - 0.98 Å and refined using the riding model approximation with U_iso_(H) = 1.2U_eq_(C).

### Crystal data

**Table:**

[Fe(C_5_H_5_)(C_32_H_23_FN_3_OS)]	*Z* = 2
*M**_r_* = 637.53	*F*(000) = 660
Triclinic, *P*1	*D*_x_ = 1.455 Mg m^−^^3^
Hall symbol: -P 1	Mo *K*α radiation, λ = 0.71073 Å
*a* = 8.7097 (2) Å	Cell parameters from 5966 reflections
*b* = 12.6456 (3) Å	θ = 1.5–26.5°
*c* = 13.5477 (4) Å	µ = 0.63 mm^−^^1^
α = 83.865 (1)°	*T* = 293 K
β = 79.008 (1)°	Block, colourless
γ = 86.776 (1)°	0.30 × 0.25 × 0.20 mm
*V* = 1455.39 (6) Å^3^	

### Data collection

**Table:**

Bruker SMART APEXII area-detector diffractometer	5966 independent reflections
Radiation source: fine-focus sealed tube	5126 reflections with *I* > 2σ(*I*)
Graphite monochromator	*R*_int_ = 0.022
ω and φ scans	θ_max_ = 26.5°, θ_min_ = 1.5°
Absorption correction: multi-scan (*SADABS*; Bruker, 2008)	*h* = −10→10
*T*_min_ = 0.833, *T*_max_ = 0.884	*k* = −15→15
22064 measured reflections	*l* = −16→15

### Refinement

**Table:**

Refinement on *F*^2^	Primary atom site location: structure-invariant direct methods
Least-squares matrix: full	Secondary atom site location: difference Fourier map
*R*[*F*^2^ > 2σ(*F*^2^)] = 0.035	Hydrogen site location: inferred from neighbouring sites
*wR*(*F*^2^) = 0.097	H-atom parameters constrained
*S* = 1.06	*w* = 1/[σ^2^(*F*_o_^2^) + (0.0495*P*)^2^ + 0.4866*P*] where *P* = (*F*_o_^2^ + 2*F*_c_^2^)/3
5966 reflections	(Δ/σ)_max_ = 0.001
397 parameters	Δρ_max_ = 0.33 e Å^−^^3^
0 restraints	Δρ_min_ = −0.34 e Å^−^^3^

### Special details

**Table:**

Geometry. All esds (except the esd in the dihedral angle between two l.s. planes) are estimated using the full covariance matrix. The cell esds are taken into account individually in the estimation of esds in distances, angles and torsion angles; correlations between esds in cell parameters are only used when they are defined by crystal symmetry. An approximate (isotropic) treatment of cell esds is used for estimating esds involving l.s. planes.
Refinement. Refinement of F^2^ against ALL reflections. The weighted R-factor wR and goodness of fit S are based on F^2^, conventional R-factors R are based on F, with F set to zero for negative F^2^. The threshold expression of F^2^ > 2sigma(F^2^) is used only for calculating R-factors(gt) etc. and is not relevant to the choice of reflections for refinement. R-factors based on F^2^ are statistically about twice as large as those based on F, and R- factors based on ALL data will be even larger.

### Fractional atomic coordinates and isotropic or equivalent isotropic displacement parameters (Å^2^)

**Table:**

	*x*	*y*	*z*	*U*_iso_*/*U*_eq_	
C1	0.2262 (2)	−0.05617 (15)	0.90688 (16)	0.0455 (5)	
C2	0.1056 (3)	−0.12677 (18)	0.9492 (2)	0.0610 (6)	
H2	0.0586	−0.1263	1.0168	0.073*	
C3	0.0579 (3)	−0.19557 (19)	0.8913 (2)	0.0676 (7)	
H3	−0.0236	−0.2404	0.9195	0.081*	
C4	0.1296 (3)	−0.19979 (19)	0.7904 (2)	0.0645 (6)	
H4	0.0975	−0.2486	0.7525	0.077*	
C5	0.2470 (3)	−0.13261 (17)	0.74675 (18)	0.0545 (5)	
H5	0.2949	−0.1363	0.6797	0.065*	
C6	0.2949 (2)	−0.05802 (14)	0.80348 (15)	0.0422 (4)	
C7	0.4407 (2)	0.08107 (14)	0.81340 (13)	0.0356 (4)	
C8	0.3754 (2)	0.08071 (14)	0.91808 (14)	0.0390 (4)	
C9	0.4426 (2)	0.16713 (15)	0.95772 (14)	0.0402 (4)	
C10	0.4181 (3)	0.19841 (19)	1.05442 (16)	0.0550 (5)	
H10	0.3498	0.1625	1.1069	0.066*	
C11	0.4977 (3)	0.2844 (2)	1.07091 (18)	0.0645 (6)	
H11	0.4824	0.3069	1.1353	0.077*	
C12	0.5997 (3)	0.3376 (2)	0.99315 (18)	0.0595 (6)	
H12	0.6522	0.3951	1.0061	0.071*	
C13	0.6249 (2)	0.30632 (17)	0.89627 (16)	0.0476 (5)	
H13	0.6946	0.3417	0.8443	0.057*	
C14	0.5441 (2)	0.22119 (15)	0.87846 (14)	0.0376 (4)	
C15	0.5453 (2)	0.17673 (14)	0.77843 (13)	0.0347 (4)	
C16	0.4710 (2)	0.25222 (13)	0.69710 (13)	0.0323 (4)	
H16	0.3694	0.2249	0.6934	0.039*	
C17	0.5827 (2)	0.24183 (14)	0.59451 (13)	0.0346 (4)	
H17	0.6462	0.3051	0.5782	0.042*	
C18	0.6895 (2)	0.14625 (14)	0.61692 (14)	0.0368 (4)	
H18	0.6387	0.0800	0.6128	0.044*	
C19	0.8574 (2)	0.14255 (17)	0.55980 (16)	0.0468 (5)	
H19A	0.8969	0.2137	0.5426	0.056*	
H19B	0.8651	0.1082	0.4983	0.056*	
C20	0.8016 (2)	0.06956 (18)	0.75419 (16)	0.0507 (5)	
H20A	0.7472	0.0032	0.7670	0.061*	
H20B	0.8373	0.0836	0.8150	0.061*	
C21	0.4942 (2)	0.23492 (14)	0.50971 (13)	0.0355 (4)	
C22	0.3981 (2)	0.14988 (15)	0.51023 (15)	0.0432 (4)	
H22	0.3942	0.0939	0.5610	0.052*	
C23	0.3088 (2)	0.14764 (16)	0.43653 (16)	0.0472 (5)	
H23	0.2447	0.0911	0.4372	0.057*	
C24	0.3172 (2)	0.23083 (17)	0.36242 (15)	0.0452 (5)	
C25	0.4097 (2)	0.31543 (16)	0.35810 (15)	0.0443 (4)	
H25	0.4131	0.3707	0.3067	0.053*	
C26	0.4984 (2)	0.31637 (14)	0.43271 (14)	0.0394 (4)	
H26	0.5624	0.3732	0.4309	0.047*	
C27	0.4460 (2)	0.36722 (14)	0.72321 (14)	0.0350 (4)	
C28	0.3001 (2)	0.39424 (14)	0.79037 (14)	0.0368 (4)	
C29	0.1728 (2)	0.32643 (16)	0.83307 (14)	0.0435 (4)	
H29	0.1722	0.2493	0.8302	0.052*	
C30	0.0485 (3)	0.39089 (19)	0.88076 (15)	0.0517 (5)	
H30	−0.0544	0.3662	0.9153	0.062*	
C31	0.0960 (3)	0.49664 (19)	0.86815 (16)	0.0541 (5)	
H31	0.0314	0.5580	0.8921	0.065*	
C32	0.2502 (2)	0.50038 (16)	0.81311 (16)	0.0451 (5)	
H32	0.3120	0.5642	0.7933	0.054*	
C33	0.0960 (3)	0.3753 (2)	0.60022 (16)	0.0540 (5)	
H33	0.1426	0.3063	0.5813	0.065*	
C34	−0.0535 (3)	0.3921 (2)	0.65521 (18)	0.0628 (6)	
H34	−0.1308	0.3376	0.6810	0.075*	
C35	−0.0738 (3)	0.4998 (3)	0.6665 (2)	0.0795 (9)	
H35	−0.1688	0.5351	0.7013	0.095*	
C36	0.0650 (4)	0.5504 (2)	0.6174 (2)	0.0794 (9)	
H36	0.0834	0.6268	0.6118	0.095*	
C37	0.1690 (3)	0.4720 (2)	0.57671 (16)	0.0591 (6)	
H37	0.2750	0.4831	0.5380	0.071*	
N1	0.2691 (2)	0.01468 (13)	0.96630 (13)	0.0475 (4)	
N2	0.40502 (19)	0.01406 (12)	0.75560 (12)	0.0413 (4)	
N3	0.70264 (17)	0.15638 (12)	0.72110 (11)	0.0372 (3)	
O1	0.54339 (16)	0.43293 (10)	0.68911 (12)	0.0518 (4)	
F1	0.22731 (18)	0.22959 (12)	0.29030 (10)	0.0684 (4)	
Fe1	0.10914 (3)	0.44031 (2)	0.730212 (19)	0.03701 (9)	
S1	0.96553 (7)	0.06529 (5)	0.64716 (5)	0.05986 (16)	

### Atomic displacement parameters (Å^2^)

**Table:**

	*U*^11^	*U*^22^	*U*^33^	*U*^12^	*U*^13^	*U*^23^
C1	0.0461 (11)	0.0371 (10)	0.0505 (12)	−0.0021 (8)	−0.0049 (9)	0.0013 (8)
C2	0.0598 (14)	0.0514 (12)	0.0649 (15)	−0.0110 (11)	0.0037 (12)	0.0025 (11)
C3	0.0600 (14)	0.0517 (13)	0.088 (2)	−0.0202 (11)	−0.0078 (13)	0.0027 (13)
C4	0.0757 (16)	0.0484 (12)	0.0747 (17)	−0.0171 (11)	−0.0247 (14)	−0.0036 (11)
C5	0.0651 (14)	0.0446 (11)	0.0559 (13)	−0.0098 (10)	−0.0135 (11)	−0.0058 (10)
C6	0.0463 (10)	0.0338 (9)	0.0462 (11)	−0.0021 (8)	−0.0103 (9)	0.0014 (8)
C7	0.0385 (9)	0.0335 (9)	0.0338 (9)	0.0008 (7)	−0.0060 (7)	−0.0007 (7)
C8	0.0428 (10)	0.0370 (9)	0.0353 (10)	0.0016 (8)	−0.0051 (8)	−0.0006 (7)
C9	0.0431 (10)	0.0436 (10)	0.0335 (10)	0.0019 (8)	−0.0069 (8)	−0.0036 (8)
C10	0.0623 (13)	0.0670 (14)	0.0336 (11)	−0.0057 (11)	−0.0012 (10)	−0.0078 (10)
C11	0.0735 (16)	0.0840 (17)	0.0392 (12)	−0.0091 (13)	−0.0060 (11)	−0.0247 (12)
C12	0.0607 (14)	0.0718 (15)	0.0517 (13)	−0.0141 (11)	−0.0117 (11)	−0.0233 (11)
C13	0.0452 (11)	0.0581 (12)	0.0421 (11)	−0.0096 (9)	−0.0082 (9)	−0.0121 (9)
C14	0.0364 (9)	0.0425 (10)	0.0343 (9)	0.0014 (7)	−0.0073 (7)	−0.0064 (8)
C15	0.0368 (9)	0.0353 (9)	0.0316 (9)	−0.0027 (7)	−0.0046 (7)	−0.0047 (7)
C16	0.0341 (9)	0.0316 (8)	0.0320 (9)	−0.0037 (7)	−0.0065 (7)	−0.0047 (7)
C17	0.0384 (9)	0.0322 (8)	0.0328 (9)	−0.0046 (7)	−0.0040 (7)	−0.0037 (7)
C18	0.0396 (9)	0.0357 (9)	0.0344 (9)	−0.0013 (7)	−0.0034 (7)	−0.0062 (7)
C19	0.0440 (11)	0.0502 (11)	0.0438 (11)	0.0028 (9)	−0.0001 (9)	−0.0103 (9)
C20	0.0477 (12)	0.0574 (12)	0.0443 (12)	0.0095 (9)	−0.0073 (9)	−0.0013 (10)
C21	0.0388 (9)	0.0349 (9)	0.0319 (9)	−0.0011 (7)	−0.0021 (7)	−0.0078 (7)
C22	0.0546 (11)	0.0359 (9)	0.0390 (10)	−0.0070 (8)	−0.0077 (9)	−0.0031 (8)
C23	0.0526 (12)	0.0425 (10)	0.0488 (12)	−0.0091 (9)	−0.0085 (9)	−0.0135 (9)
C24	0.0493 (11)	0.0538 (11)	0.0356 (10)	0.0021 (9)	−0.0114 (8)	−0.0148 (9)
C25	0.0542 (12)	0.0428 (10)	0.0346 (10)	0.0006 (9)	−0.0071 (9)	−0.0018 (8)
C26	0.0447 (10)	0.0358 (9)	0.0366 (10)	−0.0048 (8)	−0.0031 (8)	−0.0048 (7)
C27	0.0351 (9)	0.0339 (9)	0.0383 (10)	−0.0012 (7)	−0.0113 (7)	−0.0057 (7)
C28	0.0397 (9)	0.0393 (9)	0.0336 (9)	−0.0001 (7)	−0.0112 (8)	−0.0069 (7)
C29	0.0433 (10)	0.0483 (11)	0.0363 (10)	−0.0001 (8)	−0.0041 (8)	0.0006 (8)
C30	0.0438 (11)	0.0721 (14)	0.0349 (11)	0.0040 (10)	−0.0002 (9)	−0.0019 (10)
C31	0.0577 (13)	0.0649 (14)	0.0405 (11)	0.0150 (11)	−0.0076 (10)	−0.0210 (10)
C32	0.0491 (11)	0.0451 (10)	0.0460 (11)	0.0046 (8)	−0.0153 (9)	−0.0186 (9)
C33	0.0558 (13)	0.0695 (14)	0.0418 (11)	−0.0021 (11)	−0.0155 (10)	−0.0170 (10)
C34	0.0495 (13)	0.0940 (19)	0.0493 (13)	−0.0165 (12)	−0.0147 (10)	−0.0095 (12)
C35	0.0613 (16)	0.124 (3)	0.0568 (16)	0.0405 (17)	−0.0259 (13)	−0.0215 (16)
C36	0.133 (3)	0.0550 (14)	0.0589 (16)	0.0013 (16)	−0.0489 (18)	0.0058 (12)
C37	0.0595 (13)	0.0843 (17)	0.0350 (11)	−0.0204 (12)	−0.0102 (10)	−0.0015 (11)
N1	0.0524 (10)	0.0433 (9)	0.0419 (9)	−0.0045 (7)	0.0020 (8)	0.0001 (7)
N2	0.0482 (9)	0.0367 (8)	0.0386 (9)	−0.0056 (7)	−0.0058 (7)	−0.0030 (7)
N3	0.0353 (8)	0.0419 (8)	0.0336 (8)	0.0018 (6)	−0.0052 (6)	−0.0039 (6)
O1	0.0434 (8)	0.0372 (7)	0.0726 (10)	−0.0090 (6)	−0.0001 (7)	−0.0102 (7)
F1	0.0779 (9)	0.0831 (9)	0.0541 (8)	−0.0073 (7)	−0.0334 (7)	−0.0122 (7)
Fe1	0.03496 (15)	0.04285 (16)	0.03363 (15)	0.00073 (11)	−0.00654 (11)	−0.00640 (11)
S1	0.0445 (3)	0.0693 (4)	0.0606 (4)	0.0151 (3)	−0.0026 (3)	−0.0046 (3)

### Geometric parameters (Å, º)

**Table:**

C1—N1	1.377 (3)	C20—H20A	0.9700
C1—C2	1.413 (3)	C20—H20B	0.9700
C1—C6	1.417 (3)	C21—C26	1.382 (3)
C2—C3	1.362 (4)	C21—C22	1.398 (3)
C2—H2	0.9300	C22—C23	1.381 (3)
C3—C4	1.397 (4)	C22—H22	0.9300
C3—H3	0.9300	C23—C24	1.369 (3)
C4—C5	1.369 (3)	C23—H23	0.9300
C4—H4	0.9300	C24—F1	1.365 (2)
C5—C6	1.406 (3)	C24—C25	1.365 (3)
C5—H5	0.9300	C25—C26	1.386 (3)
C6—N2	1.380 (2)	C25—H25	0.9300
C7—N2	1.301 (2)	C26—H26	0.9300
C7—C8	1.423 (3)	C27—O1	1.213 (2)
C7—C15	1.535 (2)	C27—C28	1.461 (3)
C8—N1	1.312 (2)	C28—C29	1.434 (3)
C8—C9	1.464 (3)	C28—C32	1.434 (3)
C9—C10	1.383 (3)	C28—Fe1	2.0191 (18)
C9—C14	1.397 (3)	C29—C30	1.414 (3)
C10—C11	1.382 (3)	C29—Fe1	2.025 (2)
C10—H10	0.9300	C29—H29	0.9800
C11—C12	1.382 (3)	C30—C31	1.405 (3)
C11—H11	0.9300	C30—Fe1	2.045 (2)
C12—C13	1.385 (3)	C30—H30	0.9800
C12—H12	0.9300	C31—C32	1.407 (3)
C13—C14	1.385 (3)	C31—Fe1	2.051 (2)
C13—H13	0.9300	C31—H31	0.9800
C14—C15	1.520 (2)	C32—Fe1	2.0398 (19)
C15—N3	1.464 (2)	C32—H32	0.9800
C15—C16	1.588 (2)	C33—C34	1.388 (3)
C16—C27	1.527 (2)	C33—C37	1.389 (3)
C16—C17	1.551 (2)	C33—Fe1	2.047 (2)
C16—H16	0.9800	C33—H33	0.9800
C17—C21	1.513 (2)	C34—C35	1.385 (4)
C17—C18	1.523 (3)	C34—Fe1	2.047 (2)
C17—H17	0.9800	C34—H34	0.9800
C18—N3	1.457 (2)	C35—C36	1.415 (5)
C18—C19	1.520 (3)	C35—Fe1	2.025 (2)
C18—H18	0.9800	C35—H35	0.9800
C19—S1	1.821 (2)	C36—C37	1.390 (4)
C19—H19A	0.9700	C36—Fe1	2.033 (2)
C19—H19B	0.9700	C36—H36	0.9800
C20—N3	1.444 (2)	C37—Fe1	2.047 (2)
C20—S1	1.834 (2)	C37—H37	0.9800
			
N1—C1—C2	119.1 (2)	C30—C29—C28	107.56 (18)
N1—C1—C6	122.18 (17)	C30—C29—Fe1	70.45 (12)
C2—C1—C6	118.7 (2)	C28—C29—Fe1	69.03 (10)
C3—C2—C1	120.3 (2)	C30—C29—H29	126.2
C3—C2—H2	119.9	C28—C29—H29	126.2
C1—C2—H2	119.9	Fe1—C29—H29	126.2
C2—C3—C4	120.9 (2)	C31—C30—C29	108.56 (19)
C2—C3—H3	119.6	C31—C30—Fe1	70.15 (12)
C4—C3—H3	119.6	C29—C30—Fe1	68.88 (11)
C5—C4—C3	120.5 (2)	C31—C30—H30	125.7
C5—C4—H4	119.8	C29—C30—H30	125.7
C3—C4—H4	119.8	Fe1—C30—H30	125.7
C4—C5—C6	120.0 (2)	C30—C31—C32	108.85 (19)
C4—C5—H5	120.0	C30—C31—Fe1	69.72 (12)
C6—C5—H5	120.0	C32—C31—Fe1	69.46 (11)
N2—C6—C5	118.71 (19)	C30—C31—H31	125.6
N2—C6—C1	121.64 (18)	C32—C31—H31	125.6
C5—C6—C1	119.62 (18)	Fe1—C31—H31	125.6
N2—C7—C8	123.79 (17)	C31—C32—C28	107.73 (19)
N2—C7—C15	125.82 (16)	C31—C32—Fe1	70.31 (12)
C8—C7—C15	110.17 (15)	C28—C32—Fe1	68.53 (10)
N1—C8—C7	123.68 (18)	C31—C32—H32	126.1
N1—C8—C9	127.97 (18)	C28—C32—H32	126.1
C7—C8—C9	108.30 (16)	Fe1—C32—H32	126.1
C10—C9—C14	121.14 (19)	C34—C33—C37	109.0 (2)
C10—C9—C8	130.35 (19)	C34—C33—Fe1	70.18 (13)
C14—C9—C8	108.51 (16)	C37—C33—Fe1	70.17 (13)
C11—C10—C9	118.2 (2)	C34—C33—H33	125.5
C11—C10—H10	120.9	C37—C33—H33	125.5
C9—C10—H10	120.9	Fe1—C33—H33	125.5
C12—C11—C10	121.0 (2)	C35—C34—C33	107.6 (2)
C12—C11—H11	119.5	C35—C34—Fe1	69.27 (14)
C10—C11—H11	119.5	C33—C34—Fe1	70.16 (13)
C11—C12—C13	121.0 (2)	C35—C34—H34	126.2
C11—C12—H12	119.5	C33—C34—H34	126.2
C13—C12—H12	119.5	Fe1—C34—H34	126.2
C12—C13—C14	118.6 (2)	C34—C35—C36	108.3 (2)
C12—C13—H13	120.7	C34—C35—Fe1	70.97 (14)
C14—C13—H13	120.7	C36—C35—Fe1	69.87 (15)
C13—C14—C9	120.10 (18)	C34—C35—H35	125.9
C13—C14—C15	128.08 (17)	C36—C35—H35	125.9
C9—C14—C15	111.79 (16)	Fe1—C35—H35	125.9
N3—C15—C14	113.78 (14)	C37—C36—C35	107.2 (2)
N3—C15—C7	117.55 (15)	C37—C36—Fe1	70.63 (14)
C14—C15—C7	100.86 (14)	C35—C36—Fe1	69.30 (15)
N3—C15—C16	100.78 (13)	C37—C36—H36	126.4
C14—C15—C16	116.14 (14)	C35—C36—H36	126.4
C7—C15—C16	108.37 (14)	Fe1—C36—H36	126.4
C27—C16—C17	112.63 (14)	C33—C37—C36	107.9 (2)
C27—C16—C15	112.86 (14)	C33—C37—Fe1	70.15 (13)
C17—C16—C15	105.89 (14)	C36—C37—Fe1	69.52 (14)
C27—C16—H16	108.4	C33—C37—H37	126.0
C17—C16—H16	108.4	C36—C37—H37	126.0
C15—C16—H16	108.4	Fe1—C37—H37	126.0
C21—C17—C18	116.13 (15)	C8—N1—C1	114.09 (17)
C21—C17—C16	112.03 (14)	C7—N2—C6	114.56 (16)
C18—C17—C16	103.55 (14)	C20—N3—C18	108.64 (15)
C21—C17—H17	108.3	C20—N3—C15	120.93 (15)
C18—C17—H17	108.3	C18—N3—C15	108.06 (14)
C16—C17—H17	108.3	C28—Fe1—C29	41.54 (7)
N3—C18—C19	104.67 (15)	C28—Fe1—C35	174.75 (12)
N3—C18—C17	101.91 (14)	C29—Fe1—C35	143.28 (12)
C19—C18—C17	119.10 (16)	C28—Fe1—C36	134.60 (12)
N3—C18—H18	110.2	C29—Fe1—C36	174.29 (12)
C19—C18—H18	110.2	C35—Fe1—C36	40.83 (13)
C17—C18—H18	110.2	C28—Fe1—C32	41.38 (7)
C18—C19—S1	104.45 (14)	C29—Fe1—C32	69.28 (8)
C18—C19—H19A	110.9	C35—Fe1—C32	134.48 (12)
S1—C19—H19A	110.9	C36—Fe1—C32	110.44 (11)
C18—C19—H19B	110.9	C28—Fe1—C30	68.86 (8)
S1—C19—H19B	110.9	C29—Fe1—C30	40.67 (8)
H19A—C19—H19B	108.9	C35—Fe1—C30	113.71 (11)
N3—C20—S1	103.11 (13)	C36—Fe1—C30	144.91 (12)
N3—C20—H20A	111.1	C32—Fe1—C30	68.10 (9)
S1—C20—H20A	111.1	C28—Fe1—C33	115.19 (8)
N3—C20—H20B	111.1	C29—Fe1—C33	110.01 (9)
S1—C20—H20B	111.1	C35—Fe1—C33	66.68 (11)
H20A—C20—H20B	109.1	C36—Fe1—C33	66.88 (11)
C26—C21—C22	118.07 (17)	C32—Fe1—C33	146.57 (9)
C26—C21—C17	120.58 (16)	C30—Fe1—C33	134.19 (10)
C22—C21—C17	121.22 (16)	C28—Fe1—C37	110.62 (9)
C23—C22—C21	121.04 (18)	C29—Fe1—C37	134.77 (10)
C23—C22—H22	119.5	C35—Fe1—C37	67.38 (11)
C21—C22—H22	119.5	C36—Fe1—C37	39.85 (12)
C24—C23—C22	118.27 (18)	C32—Fe1—C37	116.09 (9)
C24—C23—H23	120.9	C30—Fe1—C37	173.50 (10)
C22—C23—H23	120.9	C33—Fe1—C37	39.68 (9)
F1—C24—C25	118.57 (19)	C28—Fe1—C34	144.67 (10)
F1—C24—C23	118.40 (19)	C29—Fe1—C34	113.39 (10)
C25—C24—C23	123.02 (19)	C35—Fe1—C34	39.76 (12)
C24—C25—C26	117.92 (18)	C36—Fe1—C34	67.59 (12)
C24—C25—H25	121.0	C32—Fe1—C34	173.03 (10)
C26—C25—H25	121.0	C30—Fe1—C34	109.37 (10)
C21—C26—C25	121.67 (17)	C33—Fe1—C34	39.65 (9)
C21—C26—H26	119.2	C37—Fe1—C34	67.06 (9)
C25—C26—H26	119.2	C28—Fe1—C31	68.63 (8)
O1—C27—C28	121.81 (16)	C29—Fe1—C31	68.35 (9)
O1—C27—C16	121.04 (16)	C35—Fe1—C31	110.11 (10)
C28—C27—C16	117.15 (15)	C36—Fe1—C31	115.37 (11)
C29—C28—C32	107.30 (17)	C32—Fe1—C31	40.23 (9)
C29—C28—C27	127.91 (16)	C30—Fe1—C31	40.13 (9)
C32—C28—C27	124.22 (17)	C33—Fe1—C31	172.76 (9)
C29—C28—Fe1	69.43 (11)	C37—Fe1—C31	146.21 (10)
C32—C28—Fe1	70.08 (11)	C34—Fe1—C31	133.81 (10)
C27—C28—Fe1	119.05 (13)	C19—S1—C20	93.34 (9)
			
N1—C1—C2—C3	178.2 (2)	C29—C28—Fe1—C30	37.83 (12)
C6—C1—C2—C3	0.5 (3)	C32—C28—Fe1—C30	−80.45 (13)
C1—C2—C3—C4	1.8 (4)	C27—C28—Fe1—C30	160.67 (16)
C2—C3—C4—C5	−1.7 (4)	C29—C28—Fe1—C33	−92.26 (13)
C3—C4—C5—C6	−0.6 (4)	C32—C28—Fe1—C33	149.46 (13)
C4—C5—C6—N2	−175.1 (2)	C27—C28—Fe1—C33	30.58 (17)
C4—C5—C6—C1	2.8 (3)	C29—C28—Fe1—C37	−135.23 (13)
N1—C1—C6—N2	−2.4 (3)	C32—C28—Fe1—C37	106.49 (14)
C2—C1—C6—N2	175.1 (2)	C27—C28—Fe1—C37	−12.39 (17)
N1—C1—C6—C5	179.65 (19)	C29—C28—Fe1—C34	−56.14 (19)
C2—C1—C6—C5	−2.8 (3)	C32—C28—Fe1—C34	−174.42 (15)
N2—C7—C8—N1	−2.1 (3)	C27—C28—Fe1—C34	66.7 (2)
C15—C7—C8—N1	172.84 (18)	C29—C28—Fe1—C31	81.04 (13)
N2—C7—C8—C9	−179.87 (17)	C27—C28—Fe1—C31	−156.12 (17)
C15—C7—C8—C9	−4.9 (2)	C30—C29—Fe1—C28	118.62 (17)
N1—C8—C9—C10	3.1 (4)	C30—C29—Fe1—C35	−58.1 (2)
C7—C8—C9—C10	−179.2 (2)	C28—C29—Fe1—C35	−176.72 (16)
N1—C8—C9—C14	−176.19 (19)	C30—C29—Fe1—C32	80.12 (14)
C7—C8—C9—C14	1.5 (2)	C28—C29—Fe1—C32	−38.50 (11)
C14—C9—C10—C11	−0.5 (3)	C28—C29—Fe1—C30	−118.62 (17)
C8—C9—C10—C11	−179.7 (2)	C30—C29—Fe1—C33	−135.60 (14)
C9—C10—C11—C12	−0.3 (4)	C28—C29—Fe1—C33	105.78 (12)
C10—C11—C12—C13	0.2 (4)	C30—C29—Fe1—C37	−173.20 (13)
C11—C12—C13—C14	0.8 (4)	C28—C29—Fe1—C37	68.19 (16)
C12—C13—C14—C9	−1.5 (3)	C30—C29—Fe1—C34	−92.93 (15)
C12—C13—C14—C15	176.3 (2)	C28—C29—Fe1—C34	148.45 (12)
C10—C9—C14—C13	1.4 (3)	C30—C29—Fe1—C31	36.85 (13)
C8—C9—C14—C13	−179.18 (18)	C28—C29—Fe1—C31	−81.77 (13)
C10—C9—C14—C15	−176.75 (19)	C34—C35—Fe1—C29	−55.0 (2)
C8—C9—C14—C15	2.6 (2)	C36—C35—Fe1—C29	−173.67 (16)
C13—C14—C15—N3	50.0 (3)	C34—C35—Fe1—C36	118.6 (2)
C9—C14—C15—N3	−132.05 (16)	C34—C35—Fe1—C32	−174.18 (14)
C13—C14—C15—C7	176.76 (19)	C36—C35—Fe1—C32	67.2 (2)
C9—C14—C15—C7	−5.2 (2)	C34—C35—Fe1—C30	−92.21 (17)
C13—C14—C15—C16	−66.4 (3)	C36—C35—Fe1—C30	149.15 (16)
C9—C14—C15—C16	111.59 (18)	C34—C35—Fe1—C33	37.56 (15)
N2—C7—C15—N3	−54.8 (2)	C36—C35—Fe1—C33	−81.07 (18)
C8—C7—C15—N3	130.35 (17)	C34—C35—Fe1—C37	80.82 (16)
N2—C7—C15—C14	−179.12 (18)	C36—C35—Fe1—C37	−37.81 (16)
C8—C7—C15—C14	6.08 (19)	C36—C35—Fe1—C34	−118.6 (2)
N2—C7—C15—C16	58.4 (2)	C34—C35—Fe1—C31	−135.45 (16)
C8—C7—C15—C16	−116.36 (16)	C36—C35—Fe1—C31	105.91 (18)
N3—C15—C16—C27	−109.51 (15)	C37—C36—Fe1—C28	65.81 (19)
C14—C15—C16—C27	13.9 (2)	C35—C36—Fe1—C28	−176.23 (15)
C7—C15—C16—C27	126.49 (15)	C37—C36—Fe1—C35	−118.0 (2)
N3—C15—C16—C17	14.15 (16)	C37—C36—Fe1—C32	106.61 (15)
C14—C15—C16—C17	137.56 (15)	C35—C36—Fe1—C32	−135.43 (17)
C7—C15—C16—C17	−109.85 (15)	C37—C36—Fe1—C30	−172.70 (15)
C27—C16—C17—C21	−98.14 (17)	C35—C36—Fe1—C30	−54.7 (3)
C15—C16—C17—C21	138.06 (14)	C37—C36—Fe1—C33	−37.41 (14)
C27—C16—C17—C18	135.97 (15)	C35—C36—Fe1—C33	80.55 (18)
C15—C16—C17—C18	12.17 (16)	C35—C36—Fe1—C37	118.0 (2)
C21—C17—C18—N3	−157.44 (14)	C37—C36—Fe1—C34	−80.57 (16)
C16—C17—C18—N3	−34.21 (16)	C35—C36—Fe1—C34	37.39 (16)
C21—C17—C18—C19	88.1 (2)	C37—C36—Fe1—C31	150.14 (14)
C16—C17—C18—C19	−148.65 (16)	C35—C36—Fe1—C31	−91.90 (18)
N3—C18—C19—S1	37.60 (16)	C31—C32—Fe1—C28	−119.23 (18)
C17—C18—C19—S1	150.55 (14)	C31—C32—Fe1—C29	−80.59 (14)
C18—C17—C21—C26	−128.77 (18)	C28—C32—Fe1—C29	38.64 (11)
C16—C17—C21—C26	112.55 (18)	C31—C32—Fe1—C35	65.5 (2)
C18—C17—C21—C22	55.4 (2)	C28—C32—Fe1—C35	−175.29 (15)
C16—C17—C21—C22	−63.2 (2)	C31—C32—Fe1—C36	105.50 (17)
C26—C21—C22—C23	−0.6 (3)	C28—C32—Fe1—C36	−135.27 (15)
C17—C21—C22—C23	175.32 (18)	C31—C32—Fe1—C30	−36.80 (13)
C21—C22—C23—C24	0.3 (3)	C28—C32—Fe1—C30	82.42 (13)
C22—C23—C24—F1	−179.06 (18)	C31—C32—Fe1—C33	−175.80 (16)
C22—C23—C24—C25	0.1 (3)	C28—C32—Fe1—C33	−56.6 (2)
F1—C24—C25—C26	179.02 (17)	C31—C32—Fe1—C37	148.64 (15)
C23—C24—C25—C26	−0.2 (3)	C28—C32—Fe1—C37	−92.14 (14)
C22—C21—C26—C25	0.5 (3)	C28—C32—Fe1—C31	119.23 (18)
C17—C21—C26—C25	−175.38 (17)	C31—C30—Fe1—C28	81.52 (14)
C24—C25—C26—C21	−0.2 (3)	C29—C30—Fe1—C28	−38.62 (12)
C17—C16—C27—O1	−25.5 (2)	C31—C30—Fe1—C29	120.15 (19)
C15—C16—C27—O1	94.4 (2)	C31—C30—Fe1—C35	−93.52 (17)
C17—C16—C27—C28	154.34 (15)	C29—C30—Fe1—C35	146.33 (16)
C15—C16—C27—C28	−85.81 (19)	C31—C30—Fe1—C36	−57.9 (2)
O1—C27—C28—C29	177.49 (19)	C29—C30—Fe1—C36	−178.00 (18)
C16—C27—C28—C29	−2.3 (3)	C31—C30—Fe1—C32	36.89 (13)
O1—C27—C28—C32	7.3 (3)	C29—C30—Fe1—C32	−83.26 (13)
C16—C27—C28—C32	−172.54 (17)	C31—C30—Fe1—C33	−173.38 (13)
O1—C27—C28—Fe1	91.9 (2)	C29—C30—Fe1—C33	66.47 (17)
C16—C27—C28—Fe1	−87.91 (17)	C31—C30—Fe1—C34	−136.17 (14)
C32—C28—C29—C30	−0.1 (2)	C29—C30—Fe1—C34	103.68 (15)
C27—C28—C29—C30	−171.60 (18)	C29—C30—Fe1—C31	−120.15 (19)
Fe1—C28—C29—C30	−60.19 (14)	C34—C33—Fe1—C28	147.72 (15)
C32—C28—C29—Fe1	60.13 (13)	C37—C33—Fe1—C28	−92.45 (16)
C27—C28—C29—Fe1	−111.41 (19)	C34—C33—Fe1—C29	102.87 (16)
C28—C29—C30—C31	0.2 (2)	C37—C33—Fe1—C29	−137.29 (15)
Fe1—C29—C30—C31	−59.09 (15)	C34—C33—Fe1—C35	−37.66 (18)
C28—C29—C30—Fe1	59.29 (13)	C37—C33—Fe1—C35	82.17 (19)
C29—C30—C31—C32	−0.3 (2)	C34—C33—Fe1—C36	−82.27 (19)
Fe1—C30—C31—C32	−58.57 (15)	C37—C33—Fe1—C36	37.56 (17)
C29—C30—C31—Fe1	58.31 (15)	C34—C33—Fe1—C32	−174.71 (17)
C30—C31—C32—C28	0.2 (2)	C37—C33—Fe1—C32	−54.9 (2)
Fe1—C31—C32—C28	−58.50 (13)	C34—C33—Fe1—C30	63.4 (2)
C30—C31—C32—Fe1	58.73 (15)	C37—C33—Fe1—C30	−176.78 (14)
C29—C28—C32—C31	−0.1 (2)	C34—C33—Fe1—C37	−119.8 (2)
C27—C28—C32—C31	171.83 (17)	C37—C33—Fe1—C34	119.8 (2)
Fe1—C28—C32—C31	59.62 (14)	C33—C37—Fe1—C28	104.99 (14)
C29—C28—C32—Fe1	−59.72 (13)	C36—C37—Fe1—C28	−136.05 (17)
C27—C28—C32—Fe1	112.22 (18)	C33—C37—Fe1—C29	63.85 (18)
C37—C33—C34—C35	−0.3 (3)	C36—C37—Fe1—C29	−177.19 (16)
Fe1—C33—C34—C35	59.40 (17)	C33—C37—Fe1—C35	−80.23 (18)
C37—C33—C34—Fe1	−59.65 (16)	C36—C37—Fe1—C35	38.72 (18)
C33—C34—C35—C36	0.2 (3)	C33—C37—Fe1—C36	−119.0 (2)
Fe1—C34—C35—C36	60.20 (18)	C33—C37—Fe1—C32	149.89 (14)
C33—C34—C35—Fe1	−59.96 (16)	C36—C37—Fe1—C32	−91.16 (18)
C34—C35—C36—C37	−0.1 (3)	C36—C37—Fe1—C33	119.0 (2)
Fe1—C35—C36—C37	60.75 (17)	C33—C37—Fe1—C34	−36.95 (15)
C34—C35—C36—Fe1	−60.88 (18)	C36—C37—Fe1—C34	82.01 (19)
C34—C33—C37—C36	0.2 (3)	C33—C37—Fe1—C31	−172.93 (16)
Fe1—C33—C37—C36	−59.49 (16)	C36—C37—Fe1—C31	−54.0 (2)
C34—C33—C37—Fe1	59.66 (16)	C35—C34—Fe1—C28	−175.39 (16)
C35—C36—C37—C33	0.0 (3)	C33—C34—Fe1—C28	−56.7 (2)
Fe1—C36—C37—C33	59.88 (16)	C35—C34—Fe1—C29	147.74 (16)
C35—C36—C37—Fe1	−59.90 (17)	C33—C34—Fe1—C29	−93.57 (16)
C7—C8—N1—C1	0.7 (3)	C33—C34—Fe1—C35	118.7 (2)
C9—C8—N1—C1	178.01 (18)	C35—C34—Fe1—C36	−38.37 (18)
C2—C1—N1—C8	−176.14 (19)	C33—C34—Fe1—C36	80.32 (18)
C6—C1—N1—C8	1.4 (3)	C35—C34—Fe1—C30	104.12 (18)
C8—C7—N2—C6	1.1 (3)	C33—C34—Fe1—C30	−137.19 (15)
C15—C7—N2—C6	−173.07 (17)	C35—C34—Fe1—C33	−118.7 (2)
C5—C6—N2—C7	178.99 (18)	C35—C34—Fe1—C37	−81.71 (18)
C1—C6—N2—C7	1.1 (3)	C33—C34—Fe1—C37	36.98 (16)
S1—C20—N3—C18	44.82 (17)	C35—C34—Fe1—C31	65.9 (2)
S1—C20—N3—C15	170.52 (13)	C33—C34—Fe1—C31	−175.40 (14)
C19—C18—N3—C20	−55.58 (19)	C30—C31—Fe1—C28	−82.14 (13)
C17—C18—N3—C20	179.74 (15)	C32—C31—Fe1—C28	38.28 (12)
C19—C18—N3—C15	171.53 (14)	C30—C31—Fe1—C29	−37.32 (13)
C17—C18—N3—C15	46.85 (17)	C32—C31—Fe1—C29	83.10 (13)
C14—C15—N3—C20	71.3 (2)	C30—C31—Fe1—C35	103.31 (17)
C7—C15—N3—C20	−46.3 (2)	C32—C31—Fe1—C35	−136.27 (16)
C16—C15—N3—C20	−163.71 (16)	C30—C31—Fe1—C36	147.40 (16)
C14—C15—N3—C18	−162.77 (14)	C32—C31—Fe1—C36	−92.18 (17)
C7—C15—N3—C18	79.71 (18)	C30—C31—Fe1—C32	−120.42 (19)
C16—C15—N3—C18	−37.75 (16)	C32—C31—Fe1—C30	120.42 (19)
C32—C28—Fe1—C29	−118.28 (16)	C30—C31—Fe1—C37	−177.60 (16)
C27—C28—Fe1—C29	122.84 (18)	C32—C31—Fe1—C37	−57.2 (2)
C29—C28—Fe1—C36	−173.88 (14)	C30—C31—Fe1—C34	64.86 (19)
C32—C28—Fe1—C36	67.84 (18)	C32—C31—Fe1—C34	−174.72 (14)
C27—C28—Fe1—C36	−51.0 (2)	C18—C19—S1—C20	−11.28 (15)
C29—C28—Fe1—C32	118.28 (16)	N3—C20—S1—C19	−17.86 (15)
C27—C28—Fe1—C32	−118.88 (19)		

### Hydrogen-bond geometry (Å, º)

**Table:**

*D*—H···*A*	*D*—H	H···*A*	*D*···*A*	*D*—H···*A*
C25—H25···O1^i^	0.93	2.54	3.212 (2)	129

Symmetry code: (i) −*x*+1, −*y*+1, −*z*+1.
